# Effect of Diglycerol Derivatives on Interfacial Adsorption and Micelle Properties of Potassium Dodecanoate

**DOI:** 10.1021/acsomega.4c10297

**Published:** 2025-03-12

**Authors:** Miki Abe, Tetsuya Ohata, Takeshi Yamada, Shiho Yada, Tomokazu Yoshimura

**Affiliations:** † Department of Chemistry, Faculty of Science, Nara Women’s University, Kitauoyanishi-machi, Nara 630-8506, Japan; ‡ Sakamoto Yakuhin Kogyo Co., Ltd., 3-1-62 Ayumino, Izumi, Osaka 594-1157, Japan; § Department of Industrial Chemistry, Faculty of Engineering, Tokyo University of Science, 6-3-1 Niijuku, Katsushika-ku, Tokyo 125-8585, Japan

## Abstract

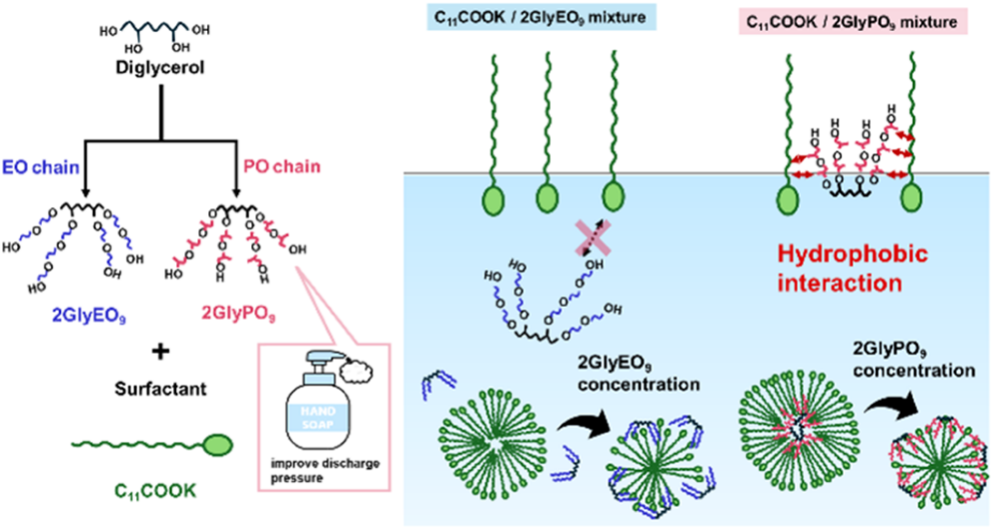

In this study, mixtures of potassium dodecanoate (C_11_COOK) and diglycerol derivatives with poly(oxyethylene) (EO) or poly(oxypropylene) (PO) chains (2GlyEO_9_ and 2GlyPO_9_) were prepared. Their adsorption behavior at the air/water interface and aggregation properties in aqueous solution were investigated by using surface tension and small-angle X-ray scattering analyses. Our observations revealed that at concentrations above the critical micelle concentration, C_11_COOK adsorbs and desorbs from the interface in C_11_COOK/2GlyPO_9_ and C_11_COOK/2GlyEO_9_ mixtures, respectively. A hydrophobic interaction was observed between the alkyl chain of C_11_COOK and the PO chain of the diglycerol derivatives in the C_11_COOK/2GlyPO_9_ mixture. However, there was no interaction between the alkyl chain of C_11_COOK and the EO chain of the diglycerol derivatives in the C_11_COOK/2GlyEO_9_ mixture. Moreover, the aggregates of the mixtures of C_11_COOK and diglycerol derivatives collapsed in aqueous solutions, regardless of the structure of the added diglycerol derivatives due to the penetration of diglycerol derivatives with EO and PO chains into the micelles or their adsorption onto the micelle surface. Therefore, different interactions occur between C_11_COOK and 2GlyEO_9_ or 2GlyPO_9,_ resulting in distinct interfacial adsorption and micelle formation behaviors, especially the difference in diglycerol derivative adducts observed in interfacial adsorption. 2GlyPO_9_ disrupts the C_11_COOK micelles in solution and improves surface properties such as static and dynamic surface tension, resulting in a lower pump pressure of pump foamers.

## Introduction

Long-chain fatty acid salts are widely used in body washes to produce creamy foams. The primary raw materials for these salts include vegetable fats, such as coconut and palm oils, and animal fats, such as beef tallow. Long-chain fatty acid salts are anionic surfactants with carboxylic acid ions in the hydrophilic group and 12–18 carbon atoms in the alkyl chain. The primary constituent of coconut-oil-based soap is dodecanoate, featuring an alkyl chain length of 12 carbon atoms. This compound is used in body washes owing to its high water solubility and excellent foaming and cleansing properties.^1,2^ Long-chain fatty acid salts are produced via saponification or neutralization methods. Saponification involves the decomposition of triglycerides into long-chain fatty acid salts and glycerol using alkaline solutions. In contrast, the neutralization method targets long-chain fatty acids.^3^ These fatty acids are obtained by decomposing fats through hydrolysis or exposure to high-pressure vapors. They are then neutralized using alkali solutions such as sodium hydroxide, potassium hydroxide (KOH), or triethanolamine to produce long-chain fatty acid salts. Due to their low dynamic surface tension, long-chain fatty acid salts exhibit excellent foamability.^4,5^ Its foamability depends on the alkyl chain, reaching the maximum at approximately C_12–14_. Both foamability and foam stability reach a maximum at the critical micelle concentration (CMC); the foam stability decreases as the concentration increases. This phenomenon was reported by Burcik,^6^ wherein the foamability increases as the rate of surface tension reduction increases. However, foam stability is achieved at an appropriate surface tension reduction rate.

Skin cleansing agents packaged in pump foamers precipitate easily, increasing the discharge pressure at low temperatures. The addition of hydrotrope-type polyols improves the discharge pressure and foam performance.^7,8^ Alcohols are polar molecules commonly used as cosolvents or cosurfactants because they exhibit a high dielectric constant and cohesive energy density, forming hydrogen bonds with water molecules. Cosolvents and cosurfactants are widely employed in pharmaceutical preparations^9^ and oil recovery^10,11^ to alter the dynamic balance of micellar systems^12^ and improve the performance of surfactants, as they are economical and environmentally friendly.

Adsorption, micelle, and foam properties have been previously studied to understand the influence of cosolvents and cosurfactants.^13−32^ Short-chain alcohols with high polarity remain in the aqueous phase as cosolvents. In contrast, long-chain alcohols with low polarity show a decreased miscibility in water and increased distribution to the micelle phase as cosurfactants.^16^ Polyols increase the solubility of hydrophobic substances in water and are used to control foamability. Aono et al. added poly(propylene glycol) (PPG)-type polyols to sodium bis(2-ethylhexyl) sulfosuccinate (AOT) aqueous solution to study the adsorption states of AOT and PPG at the air/water interface.^27^ They observed that at low concentrations, PPG decreased the AOT molecules at the interface and interacted with AOT at the interface and in the bulk. Further, foam stabilization by mixing AOT and PPG was attributed to its interfacial rheological properties because the addition of PPG changed the adsorption and orientation states of AOT at the air/water interface.

Glycerol and diglycerol-type polyols are added to improve moisture retention and viscosity-related sensory perception.^13^ A high glycerol concentration in ionic surfactant solutions leads to smaller micelle sizes and a larger CMC. This effect is attributed to an increase in curvature owing to the decrease in the dielectric constant and an increase in the electrostatic interactions.^13−15,25^ However, studies on the interfacial and aggregation properties of mixtures of surfactants and glycerol are limited, and many aspects remain unclear.

**Figure 1:**
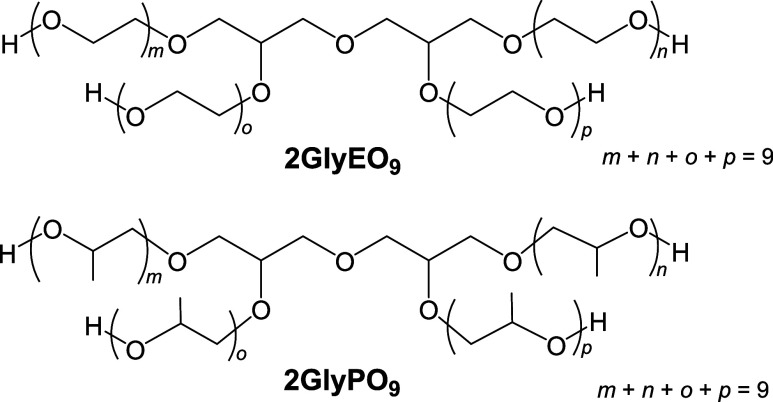
Structures of diglycerol derivatives (2GlyEO_9_ and 2GlyPO_9_).

In this study, adsorption at the air/water interface and aggregation in aqueous solution for mixtures of potassium dodecanoate (C_11_COOK) and two diglycerol derivatives of poly(oxyethylene) (EO) and poly(oxypropylene) (PO) diglyceryl ethers were investigated. EO diglyceryl ether is denoted as 2GlyEO_9_, where 2Gly is the diglycerol skeleton, EO_9_ represents the EO chains added to the hydroxy group of 2Gly, and the total average number of added moles is 9 (Figure 1). PO diglyceryl ether is represented as 2GlyPO_9_, where 2Gly is the diglycerol skeleton, PO_9_ is the PO chains added to the hydroxy group of 2Gly, and the total average number of added moles is 9 (Figure 1). This system was chosen for applications in pump foamers. The main components of the body washes used in pump foamers are higher-fatty acid salts. C_11_COOK was selected as a surfactant because it is the most widely used body wash component. However, the pump foamers have a problem with an increased discharge pressure. Because 2GlyPO_9_ is expected to reduce the discharge pressure, elucidating the physicochemical properties of a mixture with 2GlyPO_9_ would be helpful for practical use. Further, 2GlyEO_9_ is a novel compound in which an EO chain replaces the PO chain for comparison, which may provide new findings.

## Methods

### Materials

C_11_COOK was purchased from FUJIFILM Wako Pure Chemical Corporation (Osaka, Japan) and was used as received. 2GlyEO_9_, 2GlyPO_9_, and diglycerol were supplied by Sakamoto Yakuhin Kogyo Co., Ltd. (Osaka, Japan) and were used as received. The purity of the compounds (>99%) was confirmed by ^1^H NMR and ESI-MS to be free of impurities (Figures S1 and S2).

### Measurements

The static surface tensions for mixtures of C_11_COOK and diglycerol derivatives were measured by using a Krüss K100C tensiometer (Hamburg, Germany) with a resolution of 0.02 mN m^–1^, employing the Wilhelmy plate technique. The surface tension was measured at least three times with a negligible error, as can be seen in the plots. The dynamic surface tensions of the mixtures were measured on a Krüss BP2 tensiometer (Hamburg, Germany), utilizing the maximum bubble pressure technique. Each measurement was performed at least three times, and the average value was used. Two-dimensional (2D) nuclear Overhauser effect spectroscopy (NOESY) of the mixtures was performed in deuterium oxide (D_2_O) using a JEOL JNM-AL400 instrument (Tokyo, Japan) with 64 integrated cycles. Small-angle X-ray scattering (SAXS) measurements were conducted on a SAXS instrument installed at the BL40B2 beamline at SPring-8 (Hyogo, Japan). The X-ray wavelength was fixed at 0.7 Å. A large-area pixel detector (PILATUS 2M, DECTRIS Ltd., Baden) was used with a sample-to-detector distance of 2.0 m. The exposure time was 3 min. The covered *q* was within the 0.1–5 nm^–1^ range, where *q* is defined as *q* = (4π/λ) sin(θ/2) (λ and θ are the wavelength and scattering angle, respectively).

The pH of the C_11_COOK/diglycerol derivative mixtures was adjusted to 12–13, using a 0.1 mol dm^–3^ KOH solution. Potassium deuteroxide (KOD)-D_2_O solution was used for the 2D NOESY measurement. All of the measurements except 2D NOESY were performed at 25.0 ± 0.5 °C.

## Results and Discussion

### Static Surface Tension

**Figure 2:**
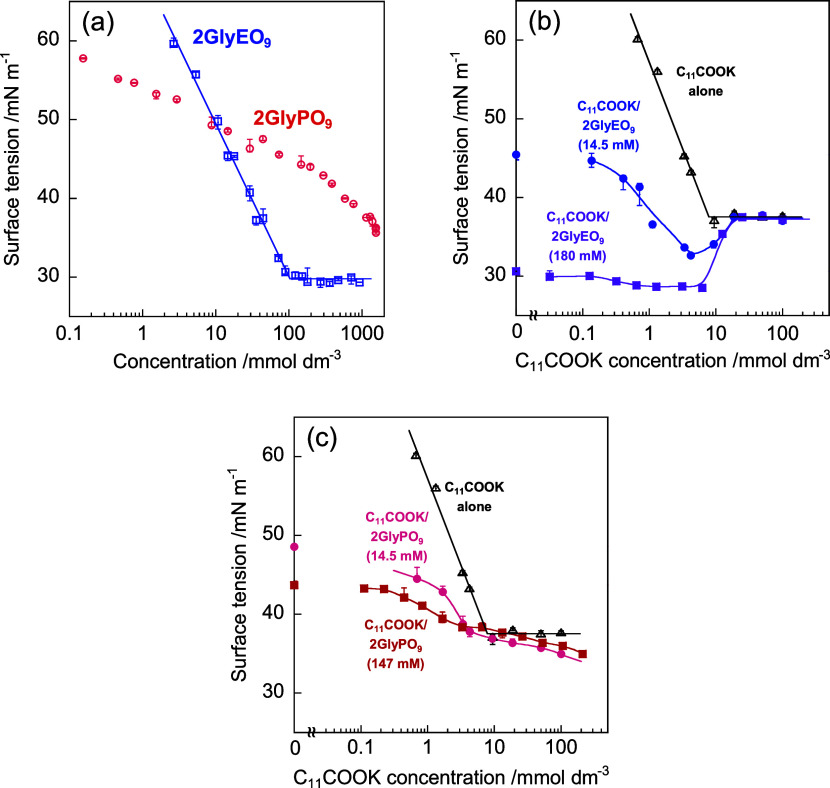
Variation in surface tension with the C_11_COOK concentration for (a) 2GlyEO_9_ and 2GlyPO_9_, (b) C_11_COOK/2GlyEO_9_ mixture, and (c) C_11_COOK/2GlyPO_9_ mixture at 25.0 ± 0.5 °C. pink open circles: 2GlyPO_9_, blue open squares: 2GlyEO_9_, open triangles: C_11_COOK, blue solid circles: C_11_COOK and 2GlyEO_9_ (14.5 mmol dm^–3^), purple solid squares: C_11_COOK and 2GlyEO_9_ (180 mmol dm^–3^), pink solid circles: C_11_COOK and 2GlyPO_9_ (14.5 mmol dm^–3^), and red solid squares: C_11_COOK and 2GlyPO_9_ (147 mmol dm^–3^). The plots with a concentration of 0 mmol dm^–3^ represent the surface tensions of the single systems of 2GlyEO_9_ and 2GlyPO_9_.

Figure 2a shows the relationship between the surface tension and concentrations of the diglycerol derivatives, namely, 2GlyEO_9_ and 2GlyPO_9_. The surface tension of 2GlyEO_9_ decreases with increasing concentration and exhibits a precise breakpoint, denoted as the CMC. This surface tension behavior is similar to that of conventional surfactants. In contrast, the surface tension of 2GlyPO_9_ decreases with increasing concentration and does not become constant even at a high concentration of 1000 mmol dm^–3^. Therefore, depending on their structures, the diglycerol derivatives with EO and PO chains exhibit different surface tension behaviors. This indicates a difference in the interfacial adsorption at the air/water interface due to the EO and PO chains. 2GlyEO_9_ formed aggregates at concentrations above 1000 mmol dm^–3^ in solution due to its amphiphilic structure comprising a diglycerol skeleton and hydrophilic EO chains and indicated a breakpoint in the surface tension plot. 2GlyPO_9_ shows continuous adsorption at the interface in multiple layers without micelle formation. It exhibits no breakpoint in the surface tension plot because it has a nonamphiphilic structure comprising a diglycerol skeleton and PO chains. The high hydrophobicity of the molecule may cause a significant decrease in surface tension at low concentrations of 2GlyPO_9_. However, a small degree of reduction in the surface tension may be due to the low surface activity. In other words, no clear CMC exists, and 2GlyPO_9_ may form the aggregates such as dimers/trimers even at the lowest concentrations studied.

Figure 2b,c illustrates the relationship between the surface tension and C_11_COOK concentration for C_11_COOK/2GlyEO_9_ and C_11_COOK/2GlyPO_9_, respectively. The surface tension behavior of the C_11_COOK/2GlyEO_9_ mixtures differs with the concentration of 2GlyEO_9_ (14.5 and 180 mmol dm^–3^). Using 14.5 mmol dm^–3^ 2GlyEO_9_, the mixture’s surface tension at low C_11_COOK concentrations is similar to that of only 2GlyEO_9_ (45.5 mN m^–1^ at 14.5 mmol dm^–3^). The surface tension of the mixture decreases with increasing C_11_COOK concentrations, reaching a minimum. It then increases by approximately 5 mN m^–1^ and, afterward, remains constant. The mixture’s constant surface tension at high C_11_COOK concentrations closely resembles that of C_11_COOK alone.

**Figure 3:**
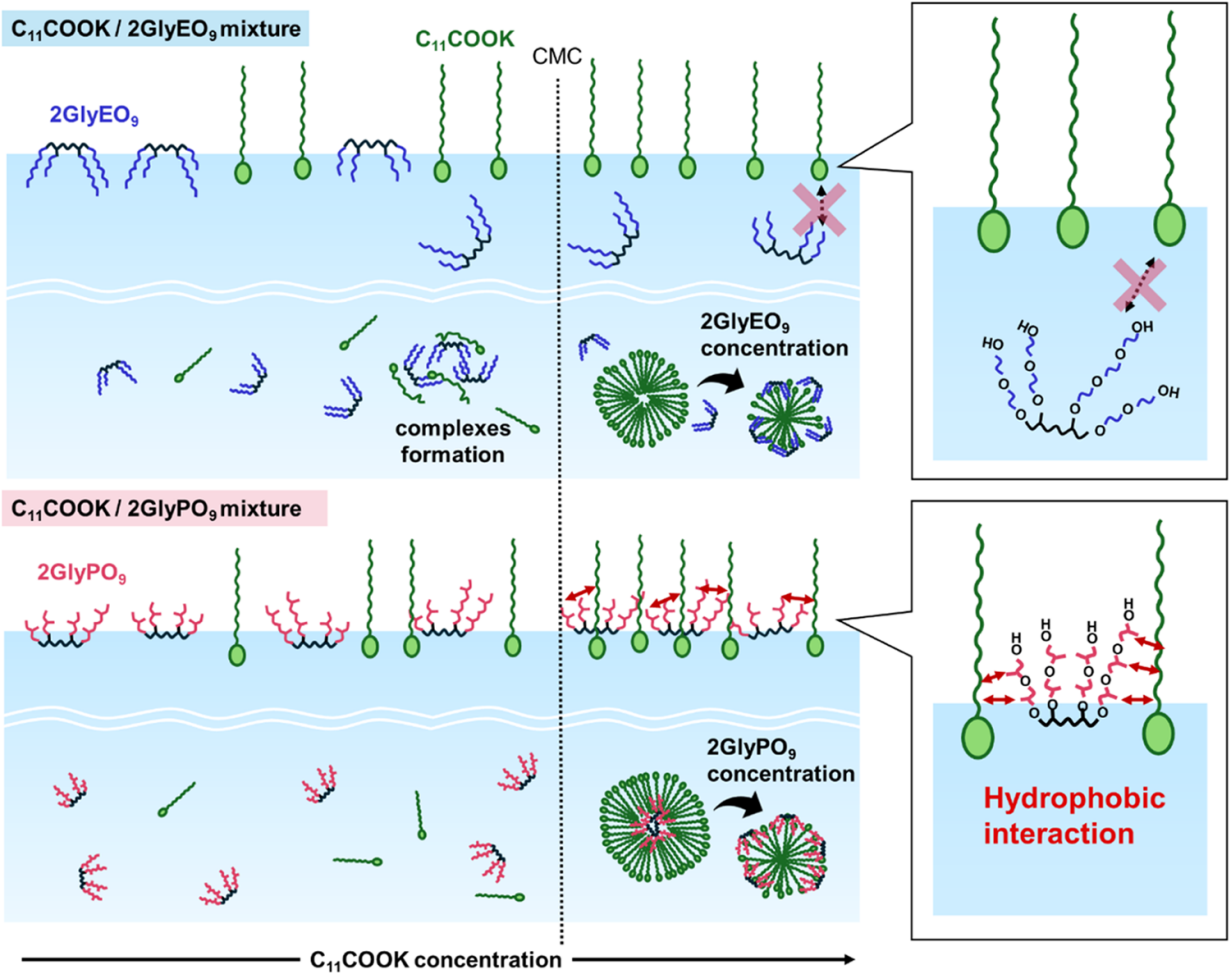
Schematic of the adsorption behavior for mixtures of C_11_COOK/2GlyEO_9_ and C_11_COOK/2GlyPO_9_.

Figure 3 shows a schematic of the adsorption behavior for mixtures of C_11_COOK/2GlyEO_9_ and C_11_COOK/2GlyPO_9_. The addition of C_11_COOK to the 2GlyEO_9_ solution results in the adsorption of both C_11_COOK and 2GlyEO_9_ at the air/water interface, and 2GlyEO_9_ starts desorbing from the interface at approximately 4 mmol dm^–3^ of C_11_COOK. However, at high C_11_COOK concentrations, only C_11_COOK adsorbs and exists at the interface. This may be because of the formation of C_11_COOK and 2GlyEO_9_ complexes due to hydrogen bonding. Penfold et al.^32^ reported that a mixture of sodium dodecyl sulfate (SDS) and polyethylenimine with EO chains formed a complex at the interface and in an aqueous solution. Moreover, they reported that the SDS molecules adsorbed at the air/water interface at low SDS concentrations decreased with increasing SDS concentration. We also previously reported that the minimum in the surface tension is caused by complex formation through hydrogen bonding, which was evidenced by adding salt to the surfactant solution.^33^ Future investigations will focus on sum-frequency generation vibrational spectroscopy (SFG) and neutron reflectivity analytical investigations to acquire detailed information about the intermolecular interaction.

In the case of 180 mmol dm^–3^ of 2GlyEO_9_, the surface tension at low C_11_COOK concentrations of the mixture (28–29 mN m^–1^ at 0.03–6.5 mmol dm^–3^ of C_11_COOK) is close to that of 2GlyEO_9_ alone (30.7 mN m^–1^ at 180 mmol dm^–3^ 2GlyEO_9_). The surface tension increases at approximately 7 mmol dm^–3^ and becomes constant at roughly the CMC of C_11_COOK alone. The constant surface tension at high C_11_COOK concentrations in the mixture is nearly identical to that of C_11_COOK alone, inferring that 2GlyEO_9_ alone or a mixture of C_11_COOK and 2GlyEO_9_ adsorbs at the air/water interface at low C_11_COOK concentrations. In contrast, only C_11_COOK adsorbs at high C_11_COOK concentrations. Therefore, the surface tension behavior of the C_11_COOK/2GlyEO_9_ mixture is affected by the 2GlyEO_9_ concentration at low C_11_COOK concentrations. However, the adsorption behavior is the same regardless of the 2GlyEO_9_ concentration at high C_11_COOK concentrations when considering the surface tension of 2GlyEO_9_ alone.

One should note that the surface tension behavior of the mixture of C_11_COOK and 2GlyEO_9_ is divided into three regions for 2GlyEO_9_ concentrations of 14.5 and 180 mmol dm^–3^. In the first region of low surfactant concentrations, intermolecular interaction occurs slightly and has only a minor effect on the surface activities of the surfactants. In the second region, at concentrations close to CMC, aggregates are formed, and C_11_COOK and 2GlyEO_9_ interact significantly at the air/water interface, resulting in a significant change in the surface tension. In the third region, at high concentrations above the CMC, C_11_COOK saturates at the interface and forms mixed micelles in the bulk. A similar discussion has been reported for mixed aqueous solutions of the anionic surfactant of SDS and poly(oxyethylene glycol) with the EO chain (PEG).^29,31,34−38^

The surface tension of the C_11_COOK/2GlyPO_9_ mixture (14.5, 147 mmol dm^–3^) decreases with increasing the C_11_COOK concentration, reaches a breakpoint, and after that decreases gradually. The surface tensions at concentrations above this breakpoint are lower than those of C_11_COOK, inferring dense adsorption of the mixtures and efficient orientation at the air/water interface due to the interactions between C_11_COOK and 2GlyPO_9_. At concentrations lower than the breakpoint, the surface tension is low at high 2GlyPO_9_ concentrations, whereas at concentrations higher than the breakpoint, it remains high. Hence, the adsorption state at the air/water interface of the C_11_COOK/diglycerol derivative mixture depends on the chain type (EO or PO) in the diglycerol derivatives.

The addition of glycerol to surfactant solutions significantly increases the surface tension at around pH 7; however, it has little effect on surface tension at lower or higher pH in solution.^39^ When an aqueous alkaline solution of the C_11_COOK/diglycerol derivative mixtures at a pH of 13 is used, similar results are obtained, particularly for the mixture containing 2GlyEO_9_. Therefore, the diglycerol skeleton and the EO chain have no effect on the decrease in dynamic surface tension.

### Dynamic Surface Tension

**Figure 4:**
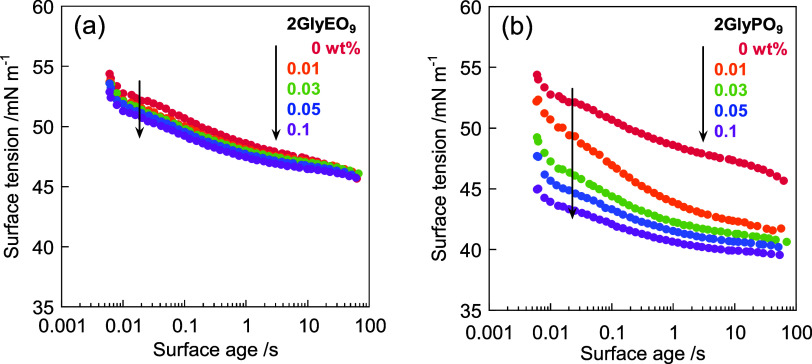
Variations in the dynamic surface tension with surface age for mixtures of C_11_COOK (0.10 wt % (4.23 mmol dm^–3^)) and (a) 2GlyEO_9_, and (b) 2GlyPO_9_ in KOH solution at 25.0 ± 0.5 °C. red solid circles: 0 wt %, orange solid circles: 0.01 wt % (2GlyEO_9_: 0.194 mmol dm^–3^, 2GlyPO_9_: 0.151 mmol dm^–3^), green solid circles: 0.03 wt % (2GlyEO_9_: 0.582 mmol dm^–3^, 2GlyPO_9_: 0.454 mmol dm^–3^), blue solid circles: 0.05 wt % (2GlyEO_9_: 0.970 mmol dm^–3^, 2GlyPO_9_: 0.756 mmol dm^–3^), and purple solid circles: 0.1 wt % (2GlyEO_9_: 1.94 mmol dm^–3^, 2GlyPO_9_: 1.51 mmol dm^–3^).

The dynamic surface tension of the C_11_COOK and diglycerol derivative mixture was measured using the maximum bubble pressure method. The adsorption dynamics at the air/water interface were also investigated. The measurements reveal that a faster decrease in surface tension results in rapid adsorption of the surfactant molecules from the bulk to the interface. Dynamic surface tension evaluates the ability of an aqueous surfactant solution to lower the surface tension instantaneously and is an essential parameter for foaming power. Figure 4 displays the relationship between surface tension and surface age for the mixtures of C_11_COOK (0.10 wt % (4.23 mmol dm^–3^), concentration below CMC), and diglycerol derivatives, 2GlyEO_9_ and 2GlyPO_9_ (0.010–0.10 wt % (2GlyEO_9_: 0.194–1.94 mmol dm^–3^, 2GlyPO_9_: 0.151–1.51 mmol dm^–3^)). C_11_COOK rapidly lowers the surface tension and instantly adsorbs at the air/water interface. The dynamic surface tension of the C_11_COOK/2GlyEO_9_ mixture is nearly similar to that of C_11_COOK, whereas that of the C_11_COOK/2GlyPO_9_ mixture decreases with increasing 2GlyPO_9_ concentration. Hence, differences in dynamic surface tension are observed depending on the adducts of the diglycerol derivatives. 2GlyPO_9_ is more hydrophobic than 2GlyEO_9_; thus, 2GlyPO_9_ and C_11_COOK rapidly adsorb at the air/water interface from the bulk, thereby lowering the surface tension. However, the decrease in surface tension for the C_11_COOK/2GlyPO_9_ mixture is at least 40 mN m^–1^. It does not exhibit the significant lowering ability observed with the static surface tension discussed earlier.

Mixtures with a C_11_COOK concentration of 5.0 wt % (222 mmol dm^–3^) above the CMC show a reduction in dynamic surface tension with the addition of 2GlyPO_9,_ analogous to that at 0.10 wt % (4.23 mmol dm^–3^) concentration (Figure S3). The PO chains of 2GlyPO_9_ affect the adsorption at the air/water interface regardless of the presence or absence of aggregates in the aqueous solution. Tan et al. reported that the addition of PPG with a PO chain to a surfactant solution decreases the dynamic surface tension.^40^ This is due to the faster adsorption rate of the surfactant molecules at the air/water interface, which is due to the higher surface activities, as the concentration of surfactant in the bulk is higher.

### 2D NOESY

**Figure 5:**
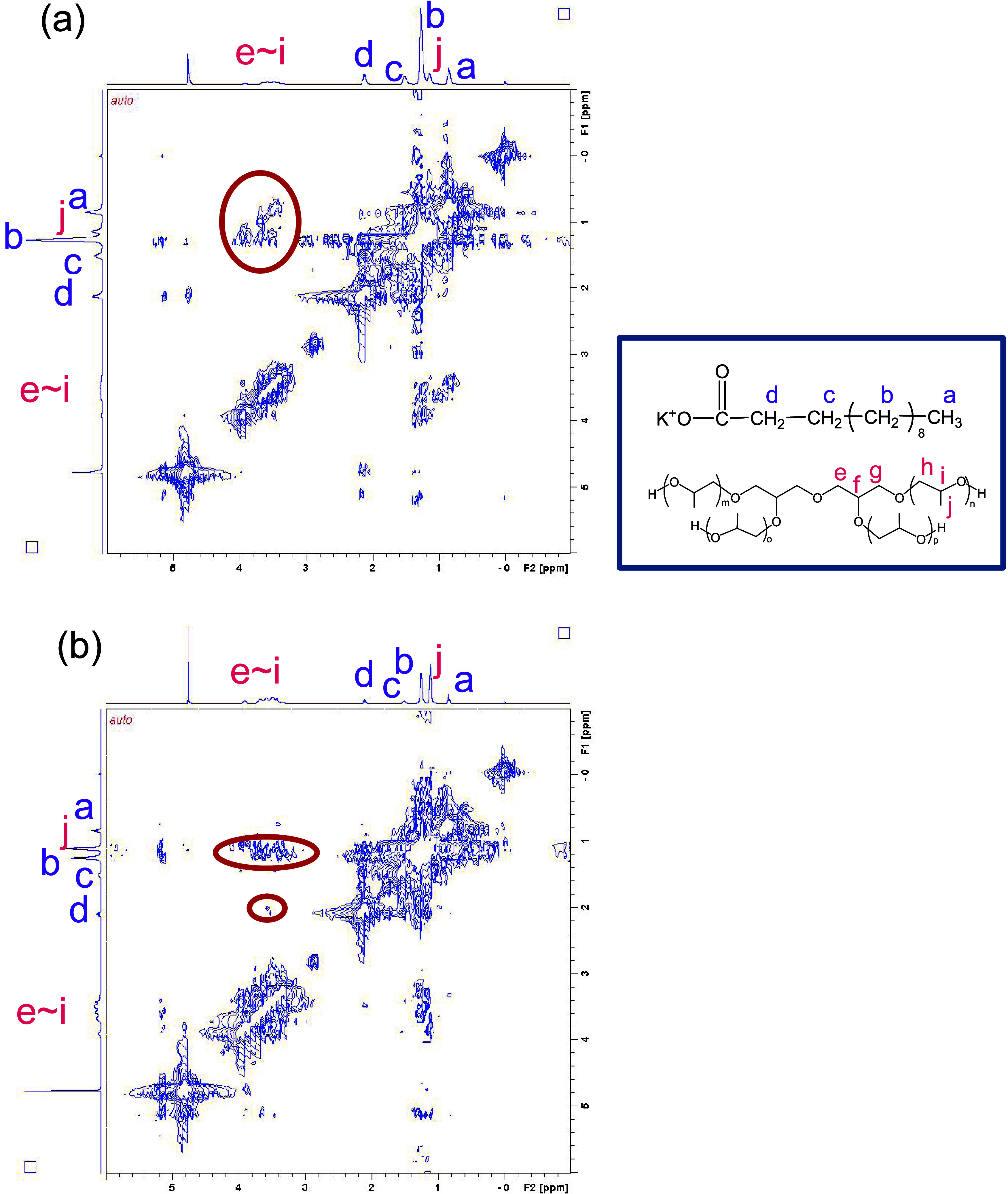
2D NOESY spectra for mixtures of C_11_COOK (5.0 wt % (232 mmol dm^–3^)) and 2GlyPO_9_ ((a) 1.0 wt % (16.0 mmol dm^–3^) and (b) 10 wt % (159 mmol dm^–3^)) in a 0.1 mol dm^–3^ D_2_O solution containing KOD.

2D NOESY is an effective method to investigate the conformation of the alkyl chain of surfactant and solubilization in micelles.^41−45^ This technique can reveal information about the spatial proximity of bound proton pairs. Protons with a spatial distance of less than 0.5 nm induce a nuclear Overhauser effect (NOE) during the mixing time of the NMR pulse sequence. There are examples of adaptation to mixed systems of surfactant and alcohol.^45^ The specific behavior of the mixture of C_11_COOK and 2GlyEO_9_ near the CMC was investigated by using 2D NOESY. Figure S4 presents the 2D NOESY spectrum of a mixture containing C_11_COOK (4.24 mmol dm^–3^) and 2GlyEO_9_ (14.5 mmol dm^–3^). A weak correlation was observed between the alkyl chains of C_11_COOK and 2GlyEO_9_, suggesting intermolecular interactions. The observed reduction in surface tension near the CMC is likely attributable to the formation of molecular complexes. Subsequently, the behavior of the mixtures at the air/water interface at concentrations exceeding the CMC was examined. Figure S5 presents the 2D NOESY spectra of mixtures containing C_11_COOK (5.0 wt % (232 mmol dm^–3^)) and 2GlyEO_9_ at concentrations of 1.0 wt % (19.0 mmol dm^–3^) and 10 wt % (194 mmol dm^–3^). Similarly, Figure 5 displays the 2D NOESY spectra of mixtures composed of C_11_COOK (5.0 wt % (232 mmol dm^–3^)) and 2GlyPO_9_ at concentrations of 1.0 wt % (16.0 mmol dm^–3^) and 10 wt % (159 mmol dm^–3^). No correlation between the two components is observed in the C_11_COOK/2GlyEO_9_ 1.0 wt % (19.0 mmol dm^–3^) mixtures. On the other hand, in the C_11_COOK/2GlyEO_9_ 10 wt % (194 mmol dm^–3^) mixture, a correlation was observed between 2GlyEO_9_ and the methylene group next to the headgroup of C_11_COOK, suggesting that 2GlyEO_9_ is solubilized in the state wrapped around the micelle. In the C_11_COOK/2GlyPO_9_ 1.0 wt % (16.0 mmol dm^–3^) mixture, cross-peaks are observed between 2GlyPO_9_ and the terminal methyl groups of C_11_COOK, indicating intermolecular interactions. The concentration of 2GlyPO_9_ is increased to 10 wt % (159 mmol dm^–3^), and the correlation between 2GlyPO_9_ and the methylene group next to the headgroup of C_11_COOK was confirmed, indicating that different concentrations of 2GlyPO_9_ show distinct intermolecular interactions. This could be attributed to an increase in the hydrophobicity of the system with increasing 2GlyPO_9_ concentrations. At low 2GlyPO_9_ concentrations, it is located inside the micelles, whereas at high concentrations, it is located on the surface of the micelles. Surface tension analysis reveals that both C_11_COOK and the diglycerol derivatives adsorb at the air/water interface. Consequently, the difference in intermolecular interactions affects the adsorption and orientation states at the air/water interface and the aggregation properties in the solution. Future investigations in our laboratory will focus on X-ray and neutron reflectivity and sum-frequency generation spectroscopy to acquire detailed information about the interactions between C_11_COOK and diglycerol derivatives at the air/water interface.

### SAXS

**Figure 6:**
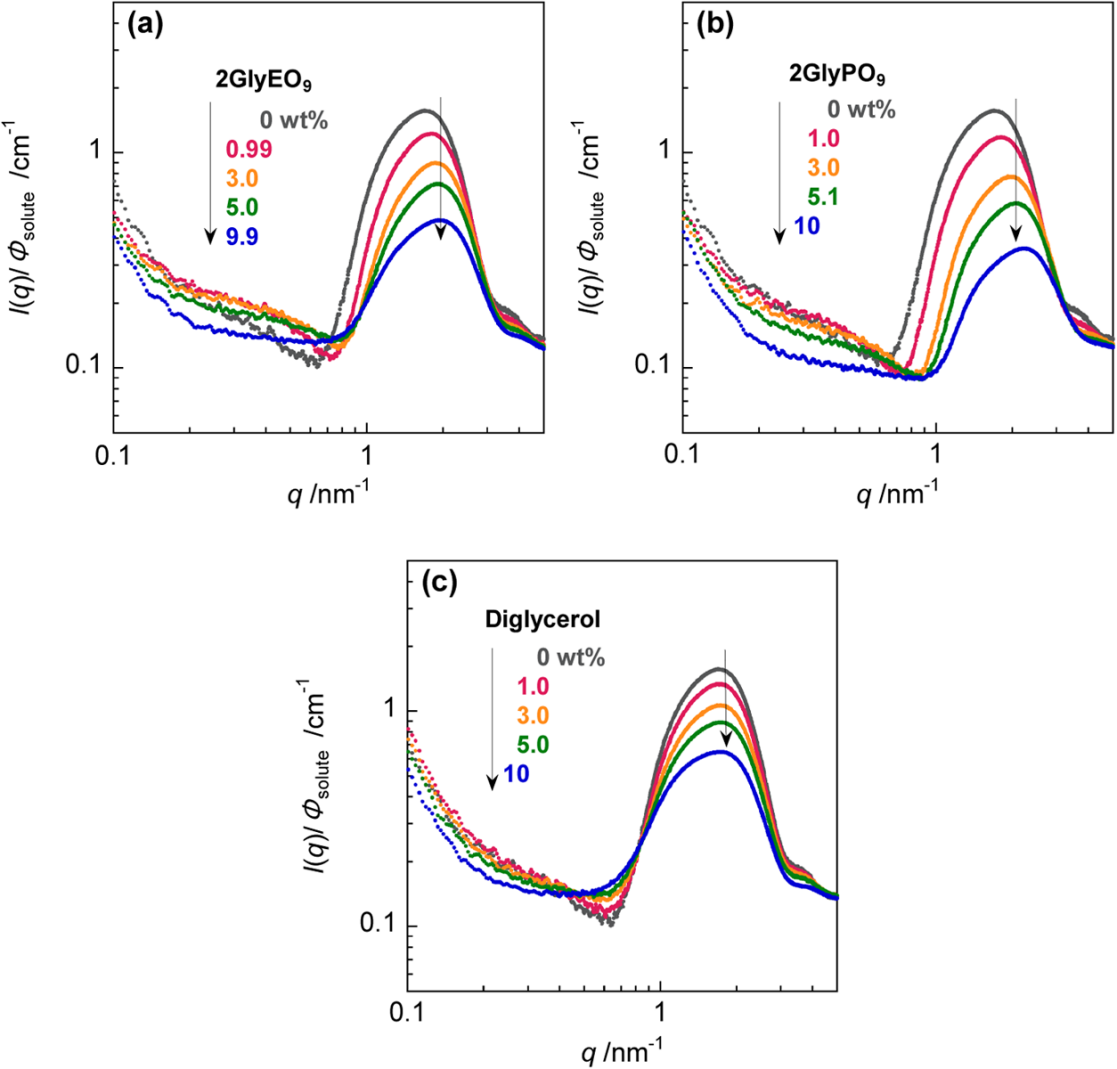
SAXS profiles for the mixtures of C_11_COOK (5.1 wt % (224.5 mmol dm^–3^)) and (a) 2GlyEO_9_, (b) 2GlyPO_9_, and (c) diglycerol in KOH solution at 25.0 ± 0.5 °C. Black solid circles: 0 wt %, pink solid circles: 1 wt % (2GlyEO_9_: 17.9 mmol dm^–3^, 2GlyPO_9_: 14.7 mmol dm^–3^, diglycerol: 64.8 mmol dm^–3^), orange solid circles: 3 wt % (2GlyEO_9_: 53.7 mmol dm^–3^, 2GlyPO_9_: 44.2 mmol dm^–3^, diglycerol: 194 mmol dm^–3^), green solid circles: 5 wt % (2GlyEO_9_: 89.5 mmol dm^–3^, 2GlyPO_9_: 73.6 mmol dm^–3^, diglycerol: 324 mmol dm^–3^), and blue solid circles: 10 wt % (2GlyEO_9_: 179 mmol dm^–3^, 2GlyPO_9_: 147 mmol dm^–3^, diglycerol: 648 mmol dm^–3^).

Figure 6 shows SAXS profiles normalized by the volume fraction φ_solute_ for mixtures of C_11_COOK (5.1 wt % (224.5 mmol dm^–3^)) and 2GlyEO_9_ or 2GlyPO_9_ (1–10 wt % (2GlyEO_9_: 17.9–179 mmol dm^–3^, 2GlyPO_9_: 14.7–147 mmol dm^–3^)), along with the mixture of C_11_COOK and diglycerol (1–10 wt % (64.8–648 mmol dm^–3^)). *q* is the magnitude of the scattering vector, and *I*(*q*) is the scattering intensity. The SAXS scattering curves exhibit a broad peak at approximately 2 nm^–1^, reflecting a difference in the electron density between the core and shell in the aggregate. The peaks of the mixtures of C_11_COOK and 2GlyEO_9_ or 2GlyPO_9_ shift to higher *q* values with increasing diglycerol derivative concentrations, whereas the peak of the mixture with diglycerol displays only a slight shift.

**Figure 7:**
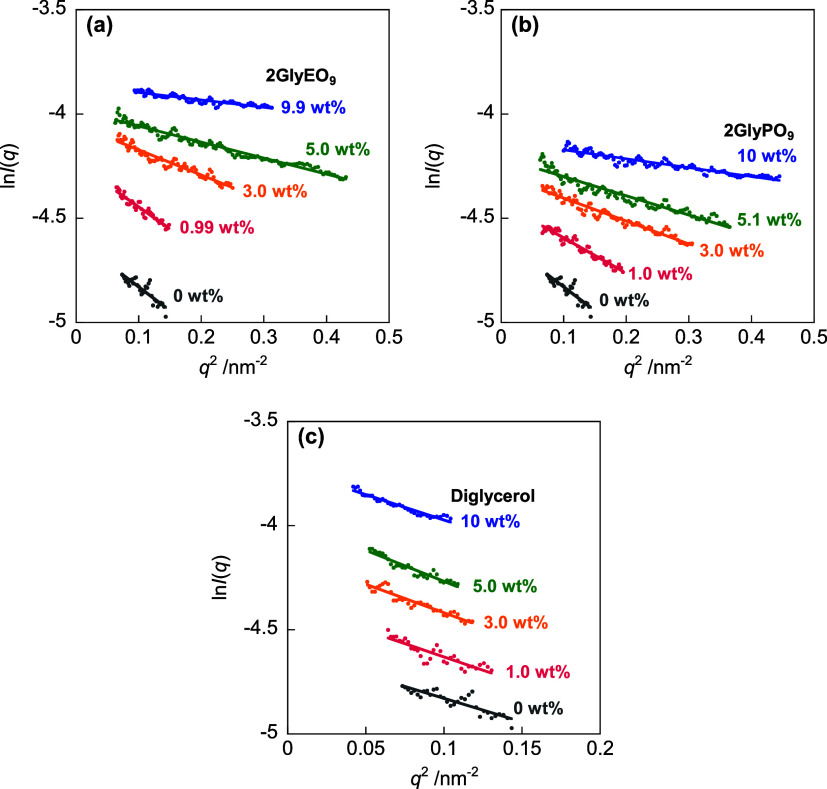
Guinier plots for mixtures of C_11_COOK (5.1 wt % (224.5 mmol dm^–3^)) and (a) 2GlyEO_9_, (b) 2GlyPO_9_, and (c) diglycerol in KOH solution at 25.0 ± 0.5 °C. Black solid circles: 0 wt %; pink solid circles: 1 wt % (2GlyEO_9_: 17.9 mmol dm^–3^, 2GlyPO_9_: 14.7 mmol dm^–3^, diglycerol: 64.8 mmol dm^–3^), orange solid circles: 3 wt % (2GlyEO_9_: 53.7 mmol dm^–3^, 2GlyPO_9_: 44.2 mmol dm^–3^, diglycerol: 194 mmol dm^–3^), green solid circles: 5 wt % (2GlyEO_9_: 89.5 mmol dm^–3^, 2GlyPO_9_: 73.6 mmol dm^–3^, diglycerol: 324 mmol dm^–3^), and blue solid circles: 10 wt % (2GlyEO_9_: 179 mmol dm^–3^, 2GlyPO_9_: 147 mmol dm^–3^, diglycerol: 648 mmol dm^–3^).

**Figure 8:**
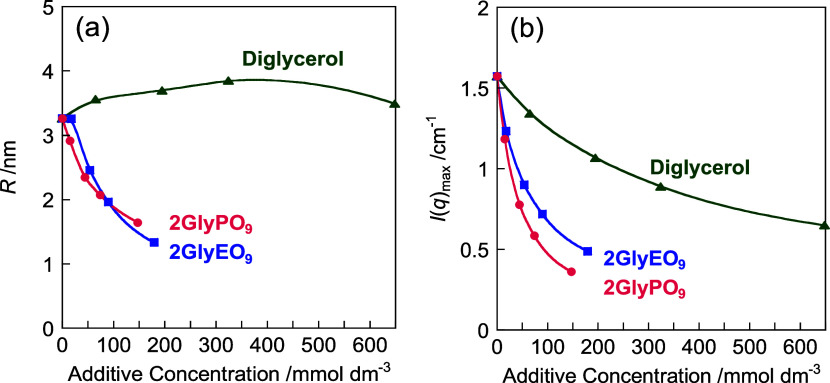
(a) Variations in the radius *R* of micelle and (b) *I*(*q*)_max_ as a function of diglycerol derivative concentration for a mixture of C_11_COOK (5 wt % (224.5 mmol dm^–3^)) and diglycerol derivatives in a KOH solution at 25.0 ± 0.5 °C. Pink solid circles: 2GlyPO_9_, blue solid squares: 2GlyEO_9_, and green solid up triangles: diglycerol.

We tried fitting the analysis using an ellipsoid model; however, the scattering profile and the theoretical curve did not match. The reason may be insufficient solvent subtraction, and this profile may reflect the high concentration of aqueous alkali solutions used in this study. The size of the micelles was estimated by using the Guinier equation as follows^46^
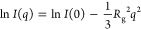
1and
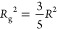
2where *R*_g_ and *R* represent the gyration radius of the micelle and the radius of the spherical micelle, respectively. Figure 7 shows the Guinier plots for the mixtures of C_11_COOK (5.1 wt % (224.5 mmol dm^–3^)) and 2GlyEO_9_ (1–10 wt % (17.9–179 mmol dm^–3^)), 2GlyPO_9_ (1–10 wt % (14.7–147 mmol dm^–3^)), and diglycerol (1–10 wt % (64.8–648 mmol dm^–3^)). The *R*_g_ value was determined using eq 1 from the slope of the linear relationship between ln *I*(*q*) and *q*^2^ in the region where *q* < 1/*R*_g_. Figure 8a displays plots of the radius of the micelles, *R*, obtained using the Guinier relation from the SAXS profile as a function of the concentration of the diglycerol derivative. In the mixture with diglycerol, *R* remains nearly identical with increasing diglycerol concentration. In contrast, *R* becomes smaller as the concentration increases in mixtures with 2GlyEO_9_ and 2GlyPO_9_. Figure 8b shows the plots of the maximum peak intensity *I*(*q*)_max_ at approximately 2 nm^–1^ vs the diglycerol derivative concentration for mixtures of C_11_COOK and diglycerol derivatives. *I*(*q*)_max_ reflects the electron density contrast between the core and shell of the micelles. In all of the systems, *I*(*q*)_max_ decreases with increasing diglycerol derivative concentrations, suggesting the collapse of the core–shell structure of the micelles. Thus, the contrast difference in electron density was reduced, possibly due to the presence of diglycerol derivatives surrounding the micelles. The micelle size of the C_11_COOK/2GlyEO_9_ mixture with the addition of 1.0 wt % (17.9 mmol dm^–3^) 2GlyEO_9_ is nearly similar to that of C_11_COOK alone. However, the size decreases at higher 2GlyEO_9_ concentrations, and at 10 wt % (179 mmol dm^–3^), it is approximately half that at 1.0 wt % (17.9 mmol dm^–3^). *I*(*q*)_max_ decreases with increasing the 2GlyEO_9_ concentration. In the C_11_COOK/2GlyPO_9_ mixture, *R* and *I*(*q*)_max_ decrease with the addition of 1.0 wt % (14.7 mmol dm^–3^) 2GlyPO_9_, inferring that the micelles collapse due to increased interactions between C_11_COOK and diglycerol derivatives as the concentration of the diglycerol derivatives increases.

*R* remains nearly constant in the C_11_COOK and diglycerol mixture, and *I*(*q*)_max_ decreases with an increase in diglycerol concentration. However, the degree of decrease is smaller than that in the mixture of 2GlyEO_9_ and 2GlyPO_9_. For this reason, diglycerol to the C_11_COOK solution resulted in a smaller electron density contrast while maintaining the micelle size. This may be because diglycerol is solubilized within the micelles but does not contribute to their collapse. In other words, the EO and PO chains of diglycerol derivatives perturb the micellar structure. This behavior is similar to that reported by Jiang et al.,^47^ who observed the collapse of worm-like micelles in a mixture where dipropylene glycol with PO chains was introduced into a mixed aqueous solution of sodium poly(oxyethylene) dodecyl ether sulfate and coconut oil fatty acid amidopropyl betaine. Furthermore, the addition of glycol to water reduces the dielectric constant and cohesive energy density, leading to the disruption of the structural order of water.^48^ Consequently, the collapse of micelles is attributed to the enhancement of electrostatic interactions between hydrophilic groups, driven by the reduction in the solvent’s dielectric constant due to glycol addition. Furthermore, the addition of a cosolvent such as glycerol to the ionic surfactant solution increases the electrostatic repulsion and curvature of the molecule, thereby decreasing the size of the aggregates.^13^ However, this behavior is not observed in the C_11_COOK/diglycerol derivative and diglycerol mixtures because dimers of glycerol or adducts of EO and PO chains to diglycerol enter the micelles or adsorb onto the surface, resulting in their collapse by disrupting the order. Adding 2GlyEO_9_ or 2GlyPO_9_ and diglycerol to C_11_COOK induces different behaviors for micelle sizes. Therefore, the diglycerol derivatives’ EO and PO chains remarkably affect the mixture’s aggregates. The water/alcohol mixture displays a high affinity for surfactants, and the micelles collapse gradually as the alcohol increases.^16^ The aforementioned 2D NOESY results indicate that diglycerol derivatives are solubilized in a state where they are wrapped around the micelles. Consequently, the EO and PO chains of the diglycerol derivatives enhanced their affinity with C_11_COOK and became solubilized around the micelle, leading to the disruption of the aggregates upon the addition of a small amount of diglycerol derivatives.

## Conclusions

The adsorption behavior of mixtures of C_11_COOK and diglycerol derivatives with EO or PO chains (2GlyEO_9_ and 2GlyPO_9_) at the air/water interface and their aggregation properties in aqueous solutions were investigated by static and dynamic surface tension, 2D NOESY, and SAXS analyses. At concentrations close to CMC, the mixture of C_11_COOK with 2GlyEO_9_ (14.5 mmol dm^–3^) exhibited the minimum surface tension. This may be due to the formation of complexes of C_11_COOK and 2GlyEO_9_. At concentrations above the CMC, the mixture with 2GlyEO_9_ showed the desorption of 2GlyEO_9_ from the interface, whereas the mixture with 2GlyPO_9_ revealed the continuous adsorption of 2GlyPO_9_ at the interface. This could be attributed to the increased hydrophobicity of the mixture of 2GlyPO_9_. Adding mixtures with diglycerol derivatives in the solution confirmed the disintegration of the aggregates. The EO and PO chains in the diglycerol derivatives showed a higher affinity for surfactants, which led to the disintegration of aggregates upon the addition of diglycerol derivatives in small quantities.

One of the main components of the body washes used in the pump foamers is C_11_COOK. C_11_COOK alone has the disadvantage of increasing the discharge pressure of the pump foamers, and the addition of 2GlyPO_9_ is expected to reduce the discharge pressure. C_11_COOK micelles in solution are disrupted by 2GlyPO_9_, which improves the surface properties (e.g., static and dynamic surface tension), resulting in a lower pump pressure of pump foamers.

## Supporting Information Available

^1^H NMR spectra of 2GlyEO_9_ and 2GlyPO_9_ (Figure S1); ESI-MS spectra of 2GlyEO_9_ and 2GlyPO_9_ (Figure S2); dynamic surface tension curves as a function of surface age for mixtures (Figure S3); 2D NOESY of the mixture of C_11_COOK (4.24 mmol dm^–3^) and 2GlyEO_9_ (14.5 mmol dm^–3^) (Figure S4); and 2D NOESY of the mixture of C_11_COOK (5.0 wt % (233 mmol dm^–3^)) and 2GlyEO_9_ 1.0 wt % (19.0 mmol dm^–3^) and 10 wt % (194 mmol dm^–3^) (Figure S5) (PDF)

## Abbreviations

CMC: critical micelle concentration
C_11_COOK: potassium dodecanoate
2GlyPO_9_: poly(oxypropylene) diglyceryl ether
2GlyEO_9_: poly(oxyethylene) diglyceryl ether
SAXS: small-angle X-ray scattering
