# Supplementary material for: Effect of Diglycerol Derivatives on Interfacial Adsorption and Micelle Properties of Potassium Dodecanoate
Source: ACS Omega. 2025 Mar 12;10(11):11231–40. doi: 10.1021/acsomega.4c10297 (PMC11947778; doi:10.1021/acsomega.4c10297)
Supplement: Supplementary file 1 — ao4c10297_si_001.pdf [file ao4c10297_si_001.pdf]

Supporting Information

Effect of Diglycerol Derivatives on Interfacial Adsorption and Micelle Properties of Potassium Dodecanoate

Miki Abe[†]*, Tetsuya Ohata*[‡]*, Takeshi Yamada*[‡]*, Shiho Yada*[§]*, and Tomokazu Yoshimura*[†,]*

[†]Department of Chemistry, Faculty of Science, Nara Women's University, Kitauoyanishi-machi, Nara 630-8506, Japan

[‡]Sakamoto Yakuhin Kogyo Co., Ltd., 3-1-62 Ayumino, Izumi, Osaka 594-1157, Japan

[§]Department of Industrial Chemistry, Faculty of Engineering, Tokyo University of Science, 6-3-1 Niijuku, Katsushika-ku, Tokyo 125-8585, Japan

*Corresponding authors Email: yoshimura@cc.nara-wu.ac.jp (T.Y.)

Contents

Figures S1. ^{1}H NMR spectra

Figures S2. ESI-MS spectra

Figures S3. Dynamic surface tension

Figures S4. 2D NOESY spectrum

Figures S5. 2D NOESY spectra

The Supporting Information includes 5 Figures on 7 pages in total.

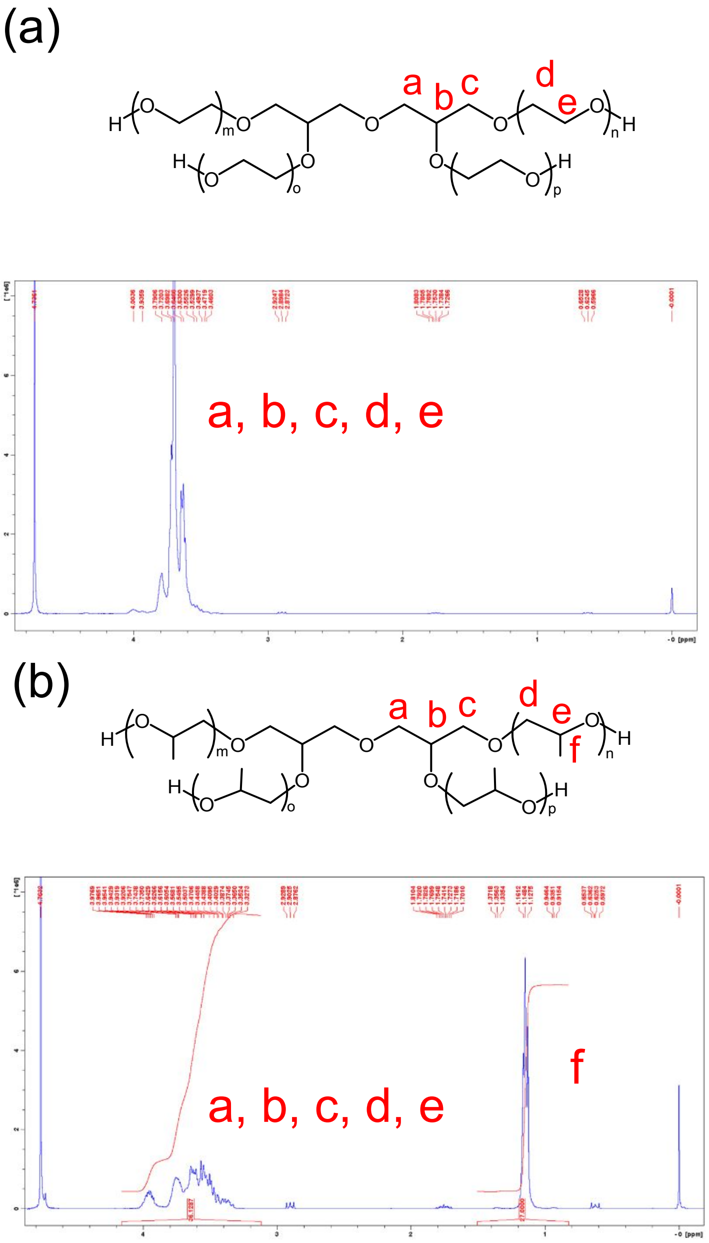

Figure S1 ^{1}H NMR spectra of (a) $2GlyEO_9$ and (b) $2GlyPO_9$.

^{1}H NMR (D_2O, TMP): **$2GlyPO_9$**: δ 1.12–1.15 (t, 27H, –CH*CH_3*–CH_2–O–) and 3.32–3.94 (m, 37H, –*CH*CH_3–*CH_2*–O–, –O–*CH_2*–*CH*–*CH_2*–O–*CH_2*–*CH*–*CH_2*–O–)
$2GlyEO_9$: δ 3.58–3.76 (m, 46H, –*CH_2*–*CH_2*–O–, –O–*CH_2*–*CH*–*CH_2*–O–*CH_2*–*CH*–*CH_2*–O–)

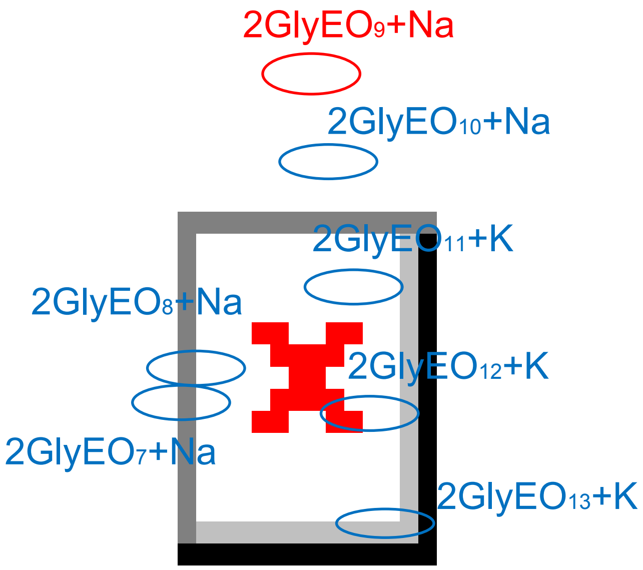

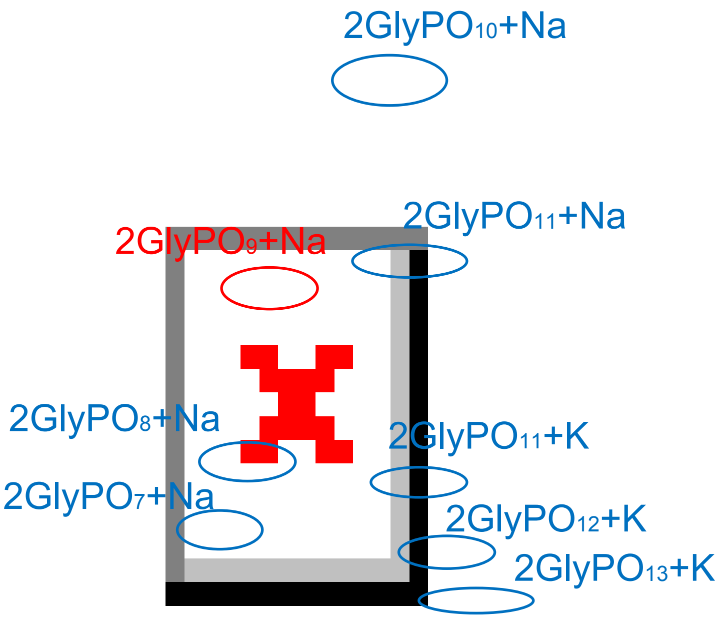

Figure S2 ESI-MS spectra of 2GlyEO$_9$ (top) and 2GlyPO$_9$ (bottom).

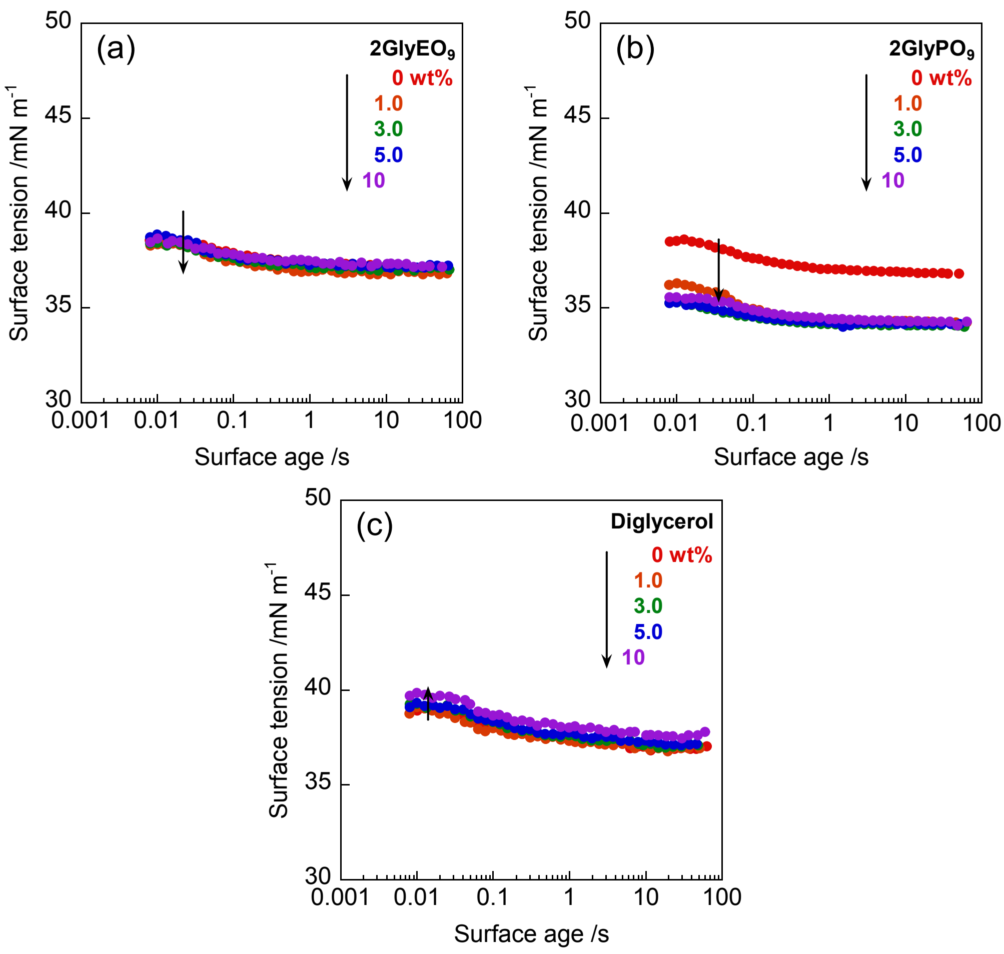

Figure S3 Dynamic surface tension of mixture as a function of surface age for mixtures of $C_{11}COOK$ (5.0 wt% (222 mmol dm^{-3})) and (a) $2GlyEO_9$, (b) $2GlyPO_9$, (c) diglycerol in KOH solution at 25.0 ± 0.5 ˚C. ●; 0 wt%, ●; 1 wt% ($2GlyEO_9$: 18.0 mmol dm^{-3}, $2GlyPO_9$: 14.5 mmol dm^{-3}, Diglycerol: 63.0 mmol dm^{-3}), ●; 3 wt% ($2GlyEO_9$: 53.9 mmol dm^{-3}, $2GlyPO_9$: 43.6 mmol dm^{-3}, Diglycerol: 189 mmol dm^{-3}), ●; 5 wt% ($2GlyEO_9$: 89.8 mmol dm^{-3}, $2GlyPO_9$: 72.7 mmol dm^{-3}, Diglycerol: 315 mmol dm^{-3}), ●; 10 wt% ($2GlyEO_9$: 180 mmol dm^{-3}, $2GlyPO_9$: 145 mmol dm^{-3}, Diglycerol: 630 mmol dm^{-3}).

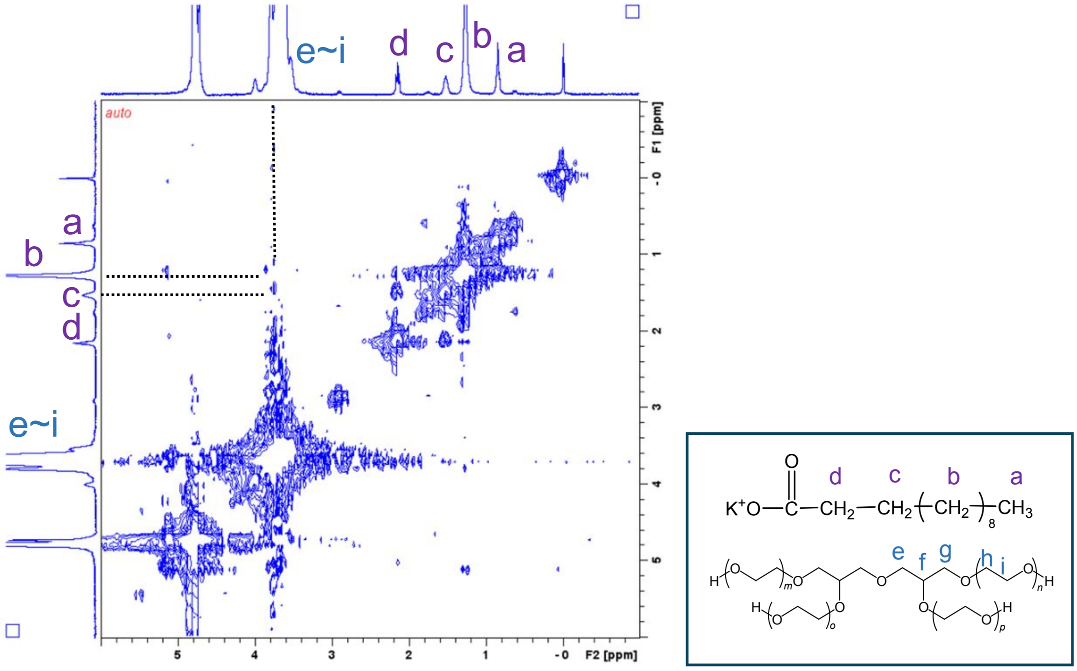

Figure S4 2D NOESY spectrum for mixtures of $C_{11}COOK$ (4.24 mmol dm^{-3}) and 2GlyEO$_9$ (14.5 mmol dm^{-3}) in 0.1 mol dm^{-3} D_2O solution containing KOD.

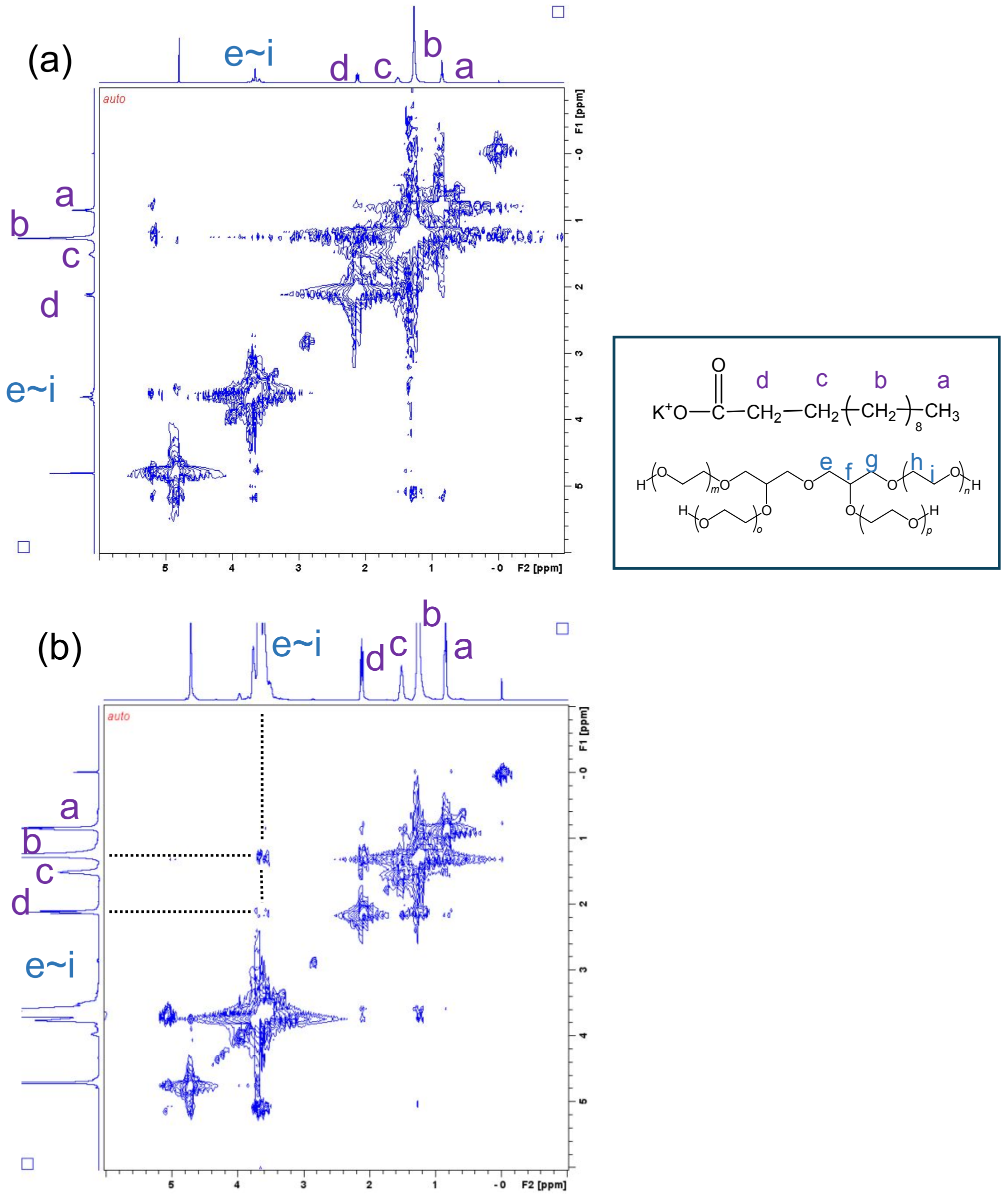

Figure S5 2D NOESY spectra of mixture of $C_{11}COOK$ (5.0 wt% (233 mmol dm^{-3})) and 2GlyEO$_9$ ((a) 1.0 wt% (19.0 mmol dm^{-3}) and (b) 10 wt% (194 mmol dm^{-3})) in 0.1 mol dm^{-3} D_2O solution containing KOD.
